# Bioactive Phenolics and Antioxidant Capacity of Some Wild Edible Greens as Affected by Different Cooking Treatments

**DOI:** 10.3390/foods9091320

**Published:** 2020-09-18

**Authors:** Lucrezia Sergio, Francesca Boari, Maria Pieralice, Vito Linsalata, Vito Cantore, Donato Di Venere

**Affiliations:** CNR-Institute of Sciences of Food Production (ISPA), Via Amendola 122/O, 70126 Bari, Italy; lucrezia.sergio@ispa.cnr.it (L.S.); francesca.boari@ispa.cnr.it (F.B.); maria.pieralice@ispa.cnr.it (M.P.); vito.linsalata@ispa.cnr.it (V.L.); vito.cantore@ispa.cnr.it (V.C.)

**Keywords:** wild species, edible herbs, processing, antioxidants, polyphenols, flavones, flavonols

## Abstract

The study aimed to assess the influence of three cooking methods (boiling, steaming, and microwave-cooking) on (i) composition in individual phenolic compounds, (ii) total phenolic content (TPC), and (iii) total antioxidant activity (TAA) of eight Mediterranean wild edible species (*Asparagus acutifolius*, *Asphodeline lutea*, *Beta vulgaris*, *Helminthotheca echioides*, *Sonchus oleraceus*, *Taraxacum officinale*, *Urospermum picroides*, *Urtica dioica*). In raw greens, several caffeic acid derivatives (chicoric, caftaric, chlorogenic, neochlorogenic, 1,5-and 3,5-dicaffeoylquinic acids) and flavonoids (glycosides of apigenin, luteolin, quercetin, isorhamnetin, kaempferol) were identified. Cooking treatments did not affect qualitative phenolic composition, while quantitative changes were recorded in some phenolic compounds and in TPC. Generally, boiling decreased TPC and TAA, while chicoric, caftaric, chlorogenic acids and quercetin-3-rutinoside increased in some species after steaming and microwave-cooking, showing positive correlation with TAA. Results confirmed steaming and microwave-cooking as mild procedures able to increase antioxidant capacity of some species, producing beneficial effects on their nutraceutical properties.

## 1. Introduction

The consumption of fruit and vegetables plays an important role in the prevention of cancer, diabetes, cardiovascular diseases, and inflammatory processes. Their antioxidative effect is mainly due to the occurrence of phenolic compounds, such as flavonoids, phenolic acids, tannins, and phenolic diterpenes. Such dietary antioxidants provide biochemical and molecular mechanisms to reduce free radicals induced by oxidative stress. Therefore, a constant supply of polyphenols is essential to provide preventive and defensive mechanisms to reduce the risk of chronic diseases in human beings [1]. The current growing interest for natural antioxidants has led to a renewed attention on wild edible greens, that are considered an important and valuable source of natural antioxidants, because of their high content in polyphenols [2,3,4], but also in minerals, organic acids, vitamins, and essential fatty acids [5,6,7]. They are interesting from an ethno-botanical point of view, since most of them are used both in traditional recipes as raw vegetables or food preparation ingredients [8] and in popular medicine as a source of alternative drugs. Recently, some of them have been considered for cultivation, and changes in biochemical parameters have been studied in relation to agronomic techniques [9,10]. Indeed, they are known as a rich source of antioxidant, anti-inflammatory, diuretic, and antimicrobial compounds [4,5,6,7,8,9,10,11,12,13,14,15,16]. Moreover, phenolic extracts from some wild edible species proved to be active against important fungal diseases during postharvest storage of some fresh fruit and vegetables [3,17]. Although the consumption of fresh vegetables is highly recommended, many wild greens are rarely consumed as raw, due to some rough sensory characteristics (e.g., pungency, bitterness, etc.), and most commonly they are eaten after cooking [8]. Nowadays, due to the growing interest in “functional foods”, consumers seek to avoid aggressive cooking methods, since they can affect nutraceutical, other than nutritional, food properties by producing heavy losses of functional phytochemicals. Moreover, it is conceivable that mild cooking methods, such as steaming and microwave-cooking (MW-cooking), which do not use large amounts of water in direct contact with the vegetable, would be more able than boiling to preserve nutritional and nutraceutical properties of greens [18]. In particular, regarding antioxidant polyphenols in wild edible greens, it might be useful to evaluate the effects of different cooking methods not only on their total content, but also on some other specific parameters (i.e., changes in the content of the pre-existing individual phenolics, disappearance of some specific compounds, and/or appearance of new phenolic compounds). Such information can help to better explain changes in antioxidant activity occurring between raw and cooked vegetables [18].

The effect of the most common domestic cooking procedures on the antioxidant activity of some wild edible species used in the Mediterranean diet has been reported [19,20]. However, as many health benefits coming from diet antioxidants have been ascribed to vegetable polyphenols ingested daily by humans [1], we believed it useful to investigate changes occurring in phenolic composition of some wild edible species due to different cooking procedures as the main cause affecting their antioxidant capacity. To the best of our knowledge, phenolic composition of species studied in the present work has been little investigated previously [8]. Furthermore, literature is lacking in findings on qualitative and quantitative changes caused by different cooking procedures on phenolic compounds of such species in relation with their antioxidant properties. Therefore, the aim of the present work was to evaluate the influence of three cooking methods (boiling, steaming, and microwave-cooking) on phenolic composition and content as related to antioxidant activity in eight Mediterranean wild edible greens (*Asparagus acutifolius* L., *Asphodeline lutea* (L.) Rchb., *Beta vulgaris* subsp. *maritima* (L.) Arcang., *Helminthotheca echioides* L., *Sonchus oleraceus* L., *Taraxacum officinale* Weber, *Urospermum picroides* (L.) Schmidt, and *Urtica dioica* (L.), collected in their own typical environment.

## 2. Materials and Methods

### 2.1. Chemicals

*Reagents*: 2,2′-azino-bis(3-ethylbenzothiazoline-6-sulfonic) acid (ABTS), ethanol (absolute, ≥99.8%), 6-hydroxy-2,5,7,8-tetramethylchromane-2-carboxylic acid (Trolox; 97%), methanol, potassium persulfate (99.0%), sodium bicarbonate (99.7%), sodium carbonate (anhydrous, 99.95%), and sodium nitrate (99.0%) were purchased from Sigma-Aldrich (Milan, Italy). Deionized water (resistivity = 18.2 MΩcm^−1^) was produced in laboratory using a Milli-Q system (Millipore, Bedford, MA, USA). All reagents were of analytical grade, except methanol (HPLC grade).

*HPLC Standards*: caffeic acid (CA), caftaric acid (caffeoyltartaric acid, CFTA), chicoric acid (dicaffeoyltartaric acid, CHCA), chlorogenic acid (5-O-caffeoylquinic acid, CHLA), 1,5-and 3,5-dicaffeoylquinic acid (1,5-and 3,5-DCQA), and neochlorogenic acid (3-O-caffeoylquinic acid, neo-CHLA) were purchased from PhytoLab GmbH & Co. KG (Vestenbergsgreuth, Germany); apigenin-7-glucoside (A-7-gluc.), isorhamnetin-3-rutinoside (I-3-rut.), kaempferol-3-rutinoside (K-3-rut.), luteolin-7-glucoside (L-7-gluc.), quercetin-3-glucoside (Q-3-gluc.), and quercetin-3-rutinoside (Q-3-rut., rutin) were purchased from Extrasynthèse (Genay, France). All HPLC standards had a chromatographic purity ≥95%.

### 2.2. Plant Material

**Figure 1:**
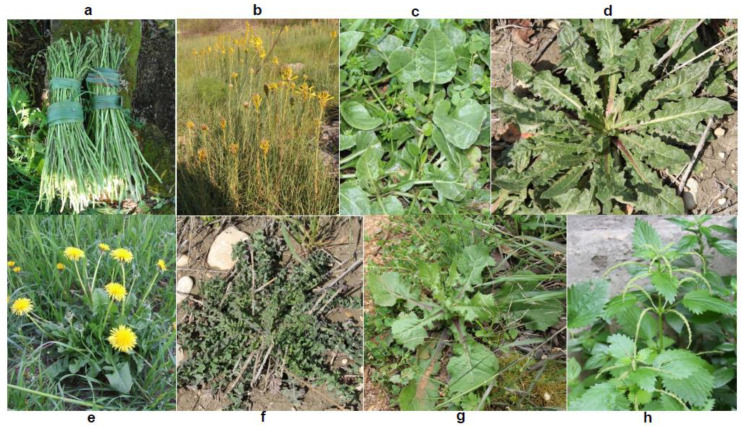
Wild greens: (**a**) *Asparagus acutifolius* L.; (**b**) *Asphodeline lutea* (L.) Rchb.; (**c**) *Beta vulgaris* subsp. maritima (L.) Arcang.; (**d**) *Helminthotheca echioides* L.; (**e**) *Sonchus oleraceus* L.; (**f**) Taraxacum officinale Weber; (**g**) *Urospermum picroides* (L.) Schmidt; (**h**) *Urtica dioica* L.

Eight wild edible greens, most commonly used in the Southern Italy and within the Mediterranean area, were selected for the present study, according to previous reports [8,19]. The selected species belonged to four botanical families, i.e., (*A. acutifolius* L. and *A. lutea* (L.) Rchb. to Liliaceae; *B. vulgaris* (L.) subsp. *maritima* (L.) Arcang. to Chenopodiaceae; *H. echioides* L., *S. oleraceus* L., *T. officinale* Weber, and *U. picroides* (L.) Schmidt to Asteraceae; *U. dioica* L. to Urticaceae) (Table 1, Figure 1).

In the aim to provide additional information regarding the antioxidant properties of these edible herbs as affected by cooking, the same species and experimental cooking protocols previously adopted by Boari et al. [19] were utilized. In particular, samples of each species were gathered, according to local consumer practices and preferences, in two consecutive years in the ‘‘Alta Murgia’’ National Park (Apulia, Southern Italy) in early spring, when plants are most suitable for eating. Plants were recognized and authenticated based on our personal knowledge as well as on the advice of botanical experts. The *A. acutifolius* and *A. lutea* spears, about 30–35 cm long, were collected when heads were still well tight and tender. Shoot apexes of *U. dioica*, about 20 cm long, were collected when still tender. Young basal and/or fully expanded leaves of *B. vulgaris*, *H. echioides*, *S. oleraceous*, *T. officinale,* and *U. picroides* were collected before flower stem appearance, by cutting the whole plant above the collar. Samples were harvested manually and a minimum of 30 plants for each species were pooled to make a single sample. Each sample was immediately preserved in a portable refrigerator and transported to the laboratory within 2 h of collecting.

### 2.3. Sample Preparation

After removing the inedible portion with a sharp steel knife, each sample was washed with tap water and then dried with a salad spinner and paper towels. Shoots of *A. lutea* were peeled before cooking, while those of *A. acutifolius* were cooked without removing the peel. The ready-to-cook samples of each species were divided into five equal portions of about 300 g, each one subdivided into three replicates of about 100 g. Two portions were kept as raw samples, the first one to be used as uncooked control and the second one to evaluate the recovery rate of the extraction procedure (see Section 2.6). The remaining three portions were cooked in triplicate, using three different cooking methods: boiling, steaming, and MW-cooking. After cooking, samples were drained-off and rapidly cooled in ice bath.

### 2.4. Cooking Conditions

Due to differences in texture characteristics among the different species, optimal cooking conditions were established according to preliminary trials, following good suggestions of the home cooking. In particular, the following cooking parameters were adopted:

- *Boiling*. Each sample was immersed in 2 L of boiling tap water in a covered stainless steel pot and cooked on electric heating plate (ARED Heating Magnetic Stirrer, Velp Scientifica, Usmate Velate, MB, Italy). Cooking times: 10 min for *B. vulgaris, H. echioides*, *S. oleraceus*; 8 min for *A. acutifolius*; 7 min for *A. lutea, T. officinale*, *U. picroides*; 3 min for *U. dioica*.
- *Steaming.* Each sample was placed in a stainless steel steam cooker, suspended in a basket, covered with a lid, and steamed with 500 mL of boiling water at atmospheric pressure using an electric heating plate (ARED Heating Magnetic Stirrer, Velp Scientifica, Usmate Velate, MB, Italy). Cooking times: 4 min for *A. acutifolius*, 3 min for *U. dioica*, and 7 min for the other species.
- *MW-cooking*. Each sample was placed in a glass pot with 200 mL of water. Pots were covered with a micro-perforated plastic film and cooked at 900 W in a commercial microwave oven (mod. MWO 112-WH, 50 Hz, Whirlpool Co., Benton Harbor, MI, USA). Cooking times were as for steaming.

### 2.5. Dry Matter Determination

Fifty grams of each replicate were weighed individually before cooking. After cooking, samples were dried in a forced draft oven at 65 °C until constant weight. Results were expressed as dry matter (DM) percentage, referring to the raw sample weight before cooking.

### 2.6. Preparation of Phenolic Extracts

Thirty grams of tissue from each replicate were homogenized and extracted (twice for 1 h) at reflux in hot water bath with boiling methanol (1:10 *w*/*v*). Raw samples to be used for recovery rate evaluation were added, before extraction, with 3.0 mg of caffeic acid (CA) per replicate as an internal standard (I.S.). The combined methanolic extracts were filtered through Whatman Grade 1 filter paper and then concentrated under reduced pressure (*P* = 20 mbar, T = 30 °C) by rotary evaporator. The residue was dissolved with a 50% (*v*/*v*) methanol/water solution and brought to a final volume of 100 mL. After subsequent filtration through Whatman Grade 1 filter paper and 0.45 µm pore size membrane filter, the solution was used for: (a) the evaluation of total phenolic concentration; (b) the HPLC analysis of phenolic compounds; and (c) the antioxidant activity assay.

### 2.7. Total Phenols

Total phenolic content (TPC) was determined by the Folin-Ciocalteu method. Briefly, a variable amount of extract (from 5 to 300 μL, depending on extract concentration) was mixed with 0.5 mL of Folin-Ciocalteu reagent, brought to the final volume of 5 mL with deionized water and gently shaken for 3 min. Then, 1 mL of sodium carbonate solution (20% *w*/*v*) was added to the reaction mixture; the solution was incubated at 40 °C for 20 min and then rapidly cooled on ice bath. Additional 4 mL of deionized water were added up to the final volume of 10 mL and then the absorbance at 750 nm was measured using a Cary 50 UV–vis spectrophotometer (Varian Inc., Palo Alto, CA, USA). CA was used as a reference standard for calibration curve; phenolic concentration was estimated as caffeic acid equivalent (CAE) and expressed as mg CAE 100 g^−1^ DM.

### 2.8. HPLC-DAD Analysis of Phenolic Compounds

**Figure 2:**
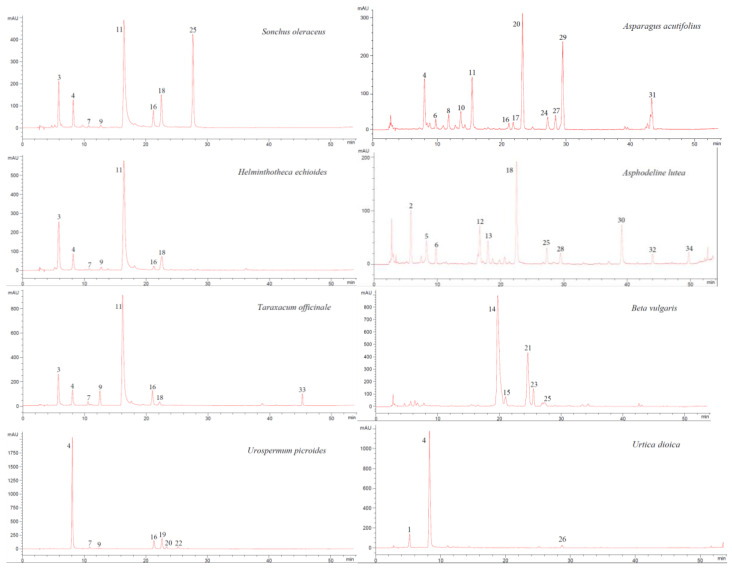
HPLC-DAD chromatograms of phenolic extracts obtained from raw wild edible herbs recorded at 325 nm. The numbering of the identified peaks is as reported in Table 2.

The HPLC analysis of phenolic compounds was performed on the same extracts used for TPC evaluation. Briefly, an Agilent 1100 Series liquid chromatograph (Agilent Technologies Inc., Santa Clara, CA, USA) equipped with a binary gradient pump (Agilent P/N G1312A), a spectrophotometric diode array detector (DAD) (Agilent P/N G1328A), and the Agilent ChemStation (Rev. A.06.03) software for spectra and data processing were used. Peak separation was achieved by a Phenomenex (Torrance, CA, USA) Luna C18 5 µm (250 mm × 4.6 mm) column, at 35 °C in thermostatic oven (Agilent P/N G1316A), and a binary gradient elution with 5% (*v*/*v*) acetic acid in deionized water (solvent A) and methanol (solvent B), at a flow rate of 1 mL min^−1^. The following elution profile was adopted: 0–25 min = 15–40% B in A; 25–30 min = isocratic 40% B in A; 30–45 min = 40–63% B in A; 45–47 min = isocratic 63% B in A; 47–52 min = 63–100% B in A; 52–56 min = isocratic 100% B, and then back to equilibrium (15% B in A). Chromatograms were recorded at three characteristic wavelengths (280, 325, and 360 nm); all chromatograms reported in Figure 2 were plotted at the same wavelength (325 nm).

The identification of the main phenolic compounds detected in the extracts was performed by comparison of chromatographic retention times as well as UV spectral data obtained by DAD with those of commercial standards. The tentative attribution of partially identified HPLC peaks as derivatives of caffeic acid (CA-der.), apigenin (A-der.), luteolin (L-der.), quercetin (Q-der.), or isorhamnetin (I-der.) was performed through UV spectral data obtained by DAD (e.g., maxima of absorbance, shape of spectra, superposition of spectra through software, etc.).

The concentration of the identified phenolic compounds was assessed using calibration curves built in the range 5–100 mg L^−1^ of each corresponding commercial standard. The correlation coefficient for calibration curves were found in all cases >0.99. The unidentified CA-der., as well as all other unidentified compounds (labelled as “unknown”), were conventionally quantified as CA. Furthermore, A-7-gluc., L-7-gluc., and Q-3-gluc. were used as reference standards for quantification of unidentified flavone and flavonol derivatives. For the reasons explained below (see Section 3.2), CA was chosen as an internal standard (I.S.) and used both to calculate the normalized retention time of each peak in the different HPLC chromatograms (Table 2), as well as to assess the percent recovery rate of the analytical method. The average percent recovery rate, calculated on raw samples, was found to be 91 ± 3%. In the experimental conditions, the CA limit of detection (LOD) and limit of quantification (LOQ), according to the IUPAC rules [21], were found to be 0.99 and 3.30 mg L^−1^, respectively. The LOQs of all the other reference standards used for peak quantification were found to be between 2.50 mg L^−1^ of A-7-gluc. and 3.75 mg L^−1^ of CHCA. The content of individual phenolic compounds was expressed as mg 100 g^−1^ DM.

### 2.9. Antioxidant Capacity

Total antioxidant activity (TAA) was assayed by the radical cation ABTS assay [22] on the same methanolic extracts used for TPC evaluation. Briefly, a 7 mM ABTS solution in deionized water was prepared. ABTS radical cation (ABTS**_•_**^+^) was produced by adding potassium persulfate to the ABTS solution up to reach a 2.45 mM final concentration and allowing the mixture to stand in the dark at room temperature for 12–16 h before use. The ABTS**^•^**^+^ solution was then diluted with absolute ethanol up to reach an absorbance of 0.70 ± 0.020 at 734 nm (blank) and thermostated at 30 °C. About 10–20 µL of extract were added in a total volume of 1 mL of ABTS**_•_**^+^ solution. The used amount varied depending on extract antioxidant capacity, in such a way as to produce a decrease of the blank absorbance in the range 20–80%. If necessary, the extract was suitably diluted with methanol to realize such a condition. The absorbance was read exactly 2.5 min after initial mixing. Antiradical activity was expressed as g Trolox 100 g^−1^ DM.

### 2.10. Statistical Analysis

Data were subjected to analysis of variance (ANOVA) using the software Prism version 6.0 (GraphPad Software Inc., San Diego, CA, USA). All determinations were made in triplicate, and data were expressed as mean ± standard deviation (SD). Mean values were separated using the Student-Newman-Keuls (SNK) test. Experiments were repeated for two consecutive years and data from the two experimental sets were combined, since the homogeneity of variances was assessed, according to Bartlett’s test.

## 3. Results and Discussion

### 3.1. Dry Matter Content

The percentage of DM in the raw wild species was as follows: *U. dioica* = 14.6 ± 2.4%; *A. lutea* = 12.9 ± 1.2%; *A. acutifolius* = 12.4 ± 0.2%; *T. officinale* = 12.2 ± 0.5%; *U. picroides* = 10.4 ± 1.7%; *B. vulgaris* = 10.3 ± 0.8%; *S. oleraceus* = 9.9 ± 2.8%; *H. echioides* = 7.2 ± 1.2%.

Each cooking procedure differently affected the DM content of the studied species. In effect, boiling caused significant decrease in DM content in all species, ranging from 32% of *U. picroides* to 7% of *A. acutifolius*. Whereas, in all species, both steaming and microwave cooking did not determine significant changes in DM content. The DM content reduction caused by boiling could be explained by the loss of soluble solids by leaching; the different extent of reduction could be attributed both to the different structural characteristics of plant tissues as well as to the different soluble solid content of each species. Actually, conventional boiling generally produces strong losses of water-soluble constituents by leaching as well as depending on the structure of phytochemicals and the different softening effect produced by the cooking process [18].

### 3.2. Phenolic Composition of Raw Wild Greens

The HPLC chromatographic profiles of phenolic extracts obtained from the eight wild species, recorded at 325 nm by UV-DAD, were reported in Figure 2. The numbering of the identified peaks in Figure 2 chromatograms was as reported in Table 2. This figure offers an immediate overview of similarities and differences in phenolic composition among the eight species.

Different classes of phenolic compounds were detected. The identity and content of phenolics detected in raw samples of each species were shown in Table 2. The maxima of absorbance shown by UV-vis spectra of the different peaks in the range 250–600 nm were also reported in Table 2. Indeed, the analysis of spectrum within this range of wavelengths is very useful for the identification of different classes of phenolic compounds, thus making the UV-vis spectrum a valid tool to assign with good reliability each phenolic compound to its own class of belonging [23].

Differently from TPC, qualitative phenolic composition of wild edible herbs is scarcely influenced by geographical and pedoclimatic factors, rather it seems to depend on genetic factors. Therefore, phenolic profiles are generally characteristic of a certain species and could be used in some cases as chemotaxonomic markers for species and sub-species identification [24,25,26,27].

Regarding the phenolic composition of the eight species studied in this work, useful information was provided by Di Venere et al. [13], Gatto et al. [3,11,17], Guarrera and Savo [8], and in other case-by-case below reported references.

Among the studied species, only the four belonging to the Asteraceae family were found to contain traces (i.e., a content below LOQ) of CA as a free compound, whereas it is absent in the other four species (Table 2). Actually, the presence of slight amounts of CA was reported in *S. oleraceus* [25], *U. picroides* [26], and *T. officinale* [28]. Moreover, CA showed a sharp chromatographic separation from the nearest peaks detected in all HPLC profiles, without overlapping with other compounds (Figure 2). Therefore, for these reasons CA was chosen as an I.S.

As shown in Table 2, several CA-der. and flavonoids were identified; moreover, some specific compounds were found in different amounts in more than one species.

As to the identified CA-der., CHLA (Figure 2, peak number 4) was detected in *A. acutifolius*, *H. echioides, S. oleraceus*, *T. officinale, U. picroides*, and *U. dioica*; CFTA (peak number 3) in *H. echioides*, *S. oleraceus,* and *T. officinale*; CHCA (peak number 11) in *A. acutifolius*, *H. echioides*, *S. oleraceus,* and *T. officinale*; 3,5-DCQA (peak number 16) in *A. acutifolius*, *H. echioides, S. oleraceus*, *T. officinale,* and *U. picroides*; finally, neo-CHLA (peak number 1) was only found in *U. dioica* (Figure 2, Table 2).

As to flavonoids, some glycosides of flavones A and L and flavonols Q, I, and K were detected. In particular, A-7-gluc. (Figure 2, peak number 25) was found in *S. oleraceus*, *A. lutea*, and *B. vulgaris*, while L-7-gluc. (peak number 18) in *S. oleraceus*, *H. echioides*, *T. officinale*, and *A. lutea*. Moreover, Q-3-gluc. (peak number 19) in *U. picroides*, Q-3-rut. (peak number 20) in *A. acutifolius* and *U. picroides*, I-3-rut. (peak number 29) and K3-rut. (peak number 27) in *A. acutifolius* were identified. Other unidentified A-, L-, Q-, and I-der. were also detected in the different species (Figure 2, Table 2).

Within three of the four species belonging to the Asteraceae family (i.e., *H. echioides*, *S. oleraceus*, and *T. officinale*), a remarkable homology among HPLC phenolic patterns was found (Figure 2). In fact, their phenolic patterns showed large amounts of CHCA (from 301 ± 42 to 725 ± 85 mg 100 g^−1^ DM), besides the presence of CFTA, CHLA, 3,5-DCQA, and L-7-gluc. Furthermore, besides the remarkable content of L-7-gluc. (444 ± 51 mg 100 g^−1^ DM), *S. oleraceus* was found to contain also a considerable amount of A-7-gluc. (544 ± 67 mg 100 g^−1^ DM) (Figure 2, Table 2). On the other hand, *U. picroides*, the fourth species belonging to the Asteraceae family, showed a different phenolic pattern, being principally rich in CHLA (1355 ± 148 mg 100 g^−1^ DM), containing a good amount of 3,5-DCQA (110 ± 13 mg 100 g^−1^ DM), and, as to flavonoids, showing the presence only of Q-der. (i.e., Q-3-gluc., Q-3-rut., and another unidentified Q-der.) (Table 2).

Concerning these four Asteraceae family species, findings on their phenolic composition resulted in good accordance with several literature reports. In particular, these species were recently investigated by Petropoulos et al. [4]; in *S. oleraceus* and *H. echioides* these authors found CHLA, CHCA, and L-7-gluc., but not CFTA and 3,5-DCQA as found in this work. On the other hand, CFTA was reported in *S. oleraceus* by Ou et al. [29], and DCQA in *H. echioides* by Giner et al. [26]. The presence of A-7-gluc. in *S. oleraceus* was also reported by Mansour et al. [24] and Giner et al. [25], in agreement with the present results. As for *T. officinale*, findings obtained in this work resulted partially consistent with Petropoulos et al. [4], which reported the presence of CHCA and L-7-gluc. only, and not also of CFTA and CHLA; on the other hand, in *T. officinale* juice, besides all components found in this work, Shütz et al. [28] reported the presence of 3,4- and 4,5-DCQA, L-7-rut., and of other unidentified L- and a Q-glycosides. As for *U. picroides*, according with the present results, Enke et al. [27] reported the presence of CHLA and 3,5-DCQA, but not of flavonoids; on the other hand Petropoulos et al. [4] found CHLA, Q-3-gluc., K-3-gluc. and other Q- and K-glycosides; while Giner et al. [26] reported the presence of CHLA, DCQA, Q-3-gluc., Q-3-galactoside (galact.), L-7-gluc., and K-3-galact.

In *A. acutifolius*, besides the presence of CHLA, CHCA, and 3,5- and 1,5- DCQA, we found remarkable amounts of Q-3-rut. and I-3-rut. (299 ± 27 and 215 ± 18 mg 100 g^−1^ DM, respectively), together with little K-3-rut. (Table 2). The same three flavonoids were found in wild asparagus by Barros et al. [2], together with a sinapic acid glycoside; but these authors did not report the presence of CHLA, CHCA and of the two DCQA found in this work. The presence of CHLA, Q-3-gluc., and Q-3-rut., but not of CHCA and I-3-rut. was also reported in this species by Di Maro et al. [30]. Moreover, according with our findings, Salvatore et al. [31] found large amounts of CA, Q, I, and K in a wild asparagus phenolic extract after acidic hydrolysis, i.e., in experimental conditions able to break glycosidic bonds and release free aglycones.

In *A. lutea*, L-7gluc. and three other unidentified L-der. were detected, together with a remarkable content of an unidentified compound (i.e., unknown 1) as well as small amounts of A-7-gluc. and of other unidentified compounds (Table 2). On the contrary, Melucci et al. [32] found the presence of rutin and other Q-der. in the flowering aerial part of *A. lutea* plants from three different origins. Such conflicting results might be possibly due to the complex anatomical composition of the aerial part of the plant, with several co-existing elements (e.g., stems, spears, flowers, etc.) which could have a different chemical composition.

In *B. vulgaris,* only flavonoids (i.e., A- and I-der.), not CA-der. were detected; in particular, two unidentified A-der. (probably two vitexin glycosides, see below) were found very abundant (1600 ± 192 and 672 ± 55 mg 100 g^−1^ DM), being the content of A-7-gluc. and of the unidentified I-der. 59 ± 8 and 122 ± 15 mg 100 g^−1^ DM, respectively (Table 2). The species *B. vulgaris* counts several tens of subspecies and varieties; actually, sugar beets and other *B. vulgaris* cultivars, such as beetroot and chard, share a common wild ancestor, the sea beet (*B. vulgaris* subsp. *maritima*). Despite the numerous literature reports on phenolic composition of the different cultivated chards, no specific information has been found on *B. vulgaris* subsp. *maritima* (L.) Arcang. However, the presence of glycosides of the flavone vitexin (i.e., apigenin-8-C-glucoside) have been reported in other *B. vulgaris* varieties. For example, in *B. vulgaris* var. *cycla*, the presence of vitexin-2-O-rhamnoside and vitexin-2-O-xyloside has been reported [33]. So, it is conceivable that the three unidentified A-der. found in this work would be vitexin glycosides, that will be deepened in further studies. However, the presence of an I-der. found in this work has never been reported in literature.

Finally, *U. dioica* showed to contain principally CHLA (563 ± 67 mg 100 g^−1^ DM), together with small amounts of neo-CHLA and of an unidentified L-der. (Table 2); in addition, traces of an unidentified A-der. were also detected. The presence of CHLA as the most abundant phenolic compound in *U. dioica* phenolic extracts was widely reported in literature [34]; besides CHLA, Carvalho et al. [35] reported the presence of 3-O-caffeoylquinic acid (i.e., neo-CHLA) in *U. dioica*, *U. urens* and *U. membranacea*. As for flavonoids, *U. dioica* was reported to contain principally Q-3-rut. [34,35]. In the present work, we found a very low flavonoid content in *U. dioica*; that might be depended on a possible low level of abiotic stress during plant growth. Indeed, it is known that the biosynthesis of flavonoids is upregulated not only as a consequence of UV-radiation but also in response to a wide range of other abiotic stresses (e.g., nitrogen/phosphorus depletion, cold, salinity/drought stress, etc.) [36]. Moreover, contrary to the literature, in *U. dioica* we found the presence of L- and A- der., which have been also reported as the main flavonoids in *U. membranacea* [30]. This might be due to a possible physiological inhibition by chelating molecules (e.g., EDTA, etc.) of flavanone-3-hydroxylase, the enzyme catalizing the hydroxylation of flavanones to dihydroflavonols [37].

### 3.3. The Effect of Cooking on Total Phenolic Content

Total phenolic content (TPC) of raw wild greens and changes caused by the different cooking methods were shown in Table 3. In the raw greens, TPC ranged from 2574 ± 247 mg CAE 100 g^−1^ DM in *U. picroides* to 735 ± 87 mg CAE 100 g^−1^ DM in *U. dioica,* with significant differences among species.

As for TPC in cooked greens, among species belonging to the Asteraceae family, no significant changes were recorded in *T. officinale*. On the contrary, TPC remarkably increased after steaming and MW-cooking in *H. echioides* (+46% and +85%, respectively) and *S. oleraceus* (+44% and +70%, respectively). Moreover, MW-cooking produced a TPC strong increase also in *U. picroides* (+80%). On the other hand, after boiling, TPC decreased in *S. oleraceus* (−54%), while it increased in *H. echioides* (+74%). Contrary to this latter finding, Savo et al. [20] reported a 50% decrease in phenolic content in 5 min boiled *H. echioides* samples and, consistently, a 75% decrease in antioxidant activity of the corresponding extract. On the contrary, according to Boari et al. [19], we found that boiling produced in *H. echioides* a slight, albeit not significant, increase in TAA, which resulted also consistent with the significant increase in TPC (Table 3). The disagreement with results reported by Savo et al. [20] might be due to different experimental conditions adopted in the two works, in particular the duration of boiling. Therefore, a higher duration of blanching applied in the present study might have promoted a greater release of phenolic compounds from intracellular proteins and altered cell wall structures, thus enhancing the availability of phenolics for extraction, up to counterbalance the loss by leaching [18].

As to remaining species, boiling produced a remarkable decrease in TPC in all of them, very strong in *U. dioica* (−75%). Steaming determined a noticeable TPC decrease only in *U. dioica* (−40%), while MW-cooking significantly affected TPC in *A. lutea*, *B. vulgaris*, and *U. dioica* (−26%, −21%, and −56%, respectively).

It was noticed that, except for *U. dioica*, steaming and MW-cooking resulted more beneficial than boiling, thus producing very low decrease or, in some cases, strong increase in TPC values with respect to the corresponding raw vegetables. Moreover, except for *H. echioides*, no data were found in literature about changes caused by cooking in phenolic content of the wild greens studied in the present work. In general, a great variability of data was observed in other species, and this can be attributed both to different cooking parameters and different vegetable matrices [18]. Furthermore, as each plant has a different pattern of phenolic compounds, it might be very useful to investigate the effect of cooking on the single phenolic compounds and/or on the different classes of phenolics.

### 3.4. The Effect of Cooking on Individual Phenolic Compounds

**Figure 3:**
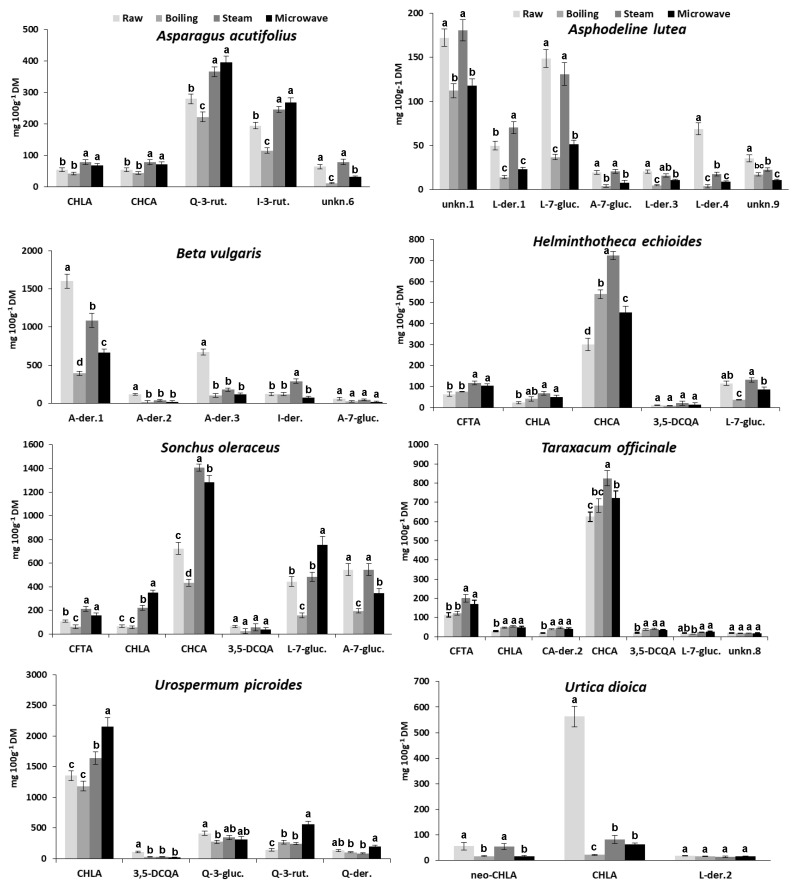
Changes in the content of the main phenolic compounds identified in wild greens depending on the different cooking treatments. Values indicate the mean ± SD (*n* = 6). Different letters indicate significant differences (*p* < 0.05) among cooking treatments for each phenolic compound in each species. (Abbreviations: CA = caffeic acid; CHLA = chlorogenic acid; CFTA = caftaric acid; CHCA = chicoric acid; DCQA = dicaffeoylquinic acid; Q = quercetin; A = apigenin; L = luteolin; I = isorhamnetin; gluc. = glucoside; rut. = rutinoside; der. = derivative; unkn. = unknown).

Changes in the content of individual phenolic compounds produced in wild greens processed by the three cooking methods were reported in Figure 3.

None of the utilized cooking procedures affected phenolic qualitative composition of the studied species. Only quantitative changes were recorded for individual phenolics due to cooking, and no new phenolic compound was detected in HPLC phenolic patterns of cooked greens, whatever the cooking method used (Figure 3). In general, results evidence a higher tendency of certain phenolic compounds to show similar behavior in different species (i.e., content increase or decrease as consequence of the same cooking treatment); nevertheless, some exceptions have been noticed.

As for hydroxycinnamic acid derivatives, the content of CHCA, CHLA, and CFTA increased by different extent after steaming as well as MW-cooking in all species in which they are present (in particular, the four species belonging to the Asteraceae family), except for *U. dioica*. On the contrary, changes in the content of these phenolic compounds proved to be different depending on the species after boiling (Figure 3). In particular, remarkable changes in CHCA content (compared with raw samples) were recorded in *H. echioides* (+79%, +140%, and +50% for boiled, steamed, and MW-cooked samples, respectively), and in *T. officinale* (+33% for steamed samples). Moreover, CHCA content remarkably increased in *S. oleraceus* steamed and MW-cooked samples (+94% and +77%, respectively), whereas it decreased (−40%) due to boiling. The CHLA content increased very strongly in steamed and MW-cooked *S. oleraceus* samples (+240% and +430%, respectively) as well as MW-cooked *U. picroides* samples (+54%). On the contrary, it drastically decreased in boiled, steamed, and MW-cooked *U. dioica* samples (−96%, −85%, and −89%, respectively). A remarkable increase in CFTA content was also recorded in steamed *S. oleraceus* and *T. officinale* (+94% and +77%, respectively) (Figure 3).

As for the behavior with cooking of the three abovementioned hydroxycinnamic acid derivatives, CHCA generally showed a great stability with all cooking methods, showing moderate or strong content increase in all the three Asteraceae species in which it is present, except in boiled *S. oleraceus*, in which it significantly decreased. This result demonstrated the great dependence on the vegetable matrix of the behavior with cooking of a single phenolic compound. In fact, it was reported that boiling for 8 min decreased TPC in stems of “Galatina” chicory, but had no effect on TPC in stems of “Molfettese” chicory, thus showing different behaviors in two local varieties of the same species (*Cichorium intybus* L., Catalogna group) [38]. As for CHLA and CFTA, a good stability with all cooking methods was observed in all species, except in the case of whatever cooked *U. dioica* samples, in which a drastic decrease in CHLA content was recorded. Also in this case, this finding could be explained invoking the matrix effect; the extreme delicacy of its leaves is very evident and, probably, shorter cooking times and a reduced volume of cooking water with respect to those adopted in this work would be advisable to avoid heavy losses of phenols and other nutrients [18], considering the great potential of application of this species in the food and feed field [34].

As for flavonoids, we found that their content remarkably decreased after boiling in most of species, whatever the aglycone present in the different compounds. On the contrary, steaming and MW-cooking showed a different impact depending on the aglycone. In fact, Q- and I-der. generally showed a content increase, contrary to L- and A-der., whose content decreased or kept quite unchanged after these latter cooking treatments, with the only exception of L-7-gluc. in the *S. oleraceus* MW-cooked samples (Figure 3).

In particular, the content of Q-3-rut. and I-3-rut. in *A. acutifolius* decreased with boiling (−26% and −46%, respectively) and increased with MW-cooking (+32%, and +25%, respectively). A remarkable increase in Q-3-rut. content was recorded in all processed *U. picroides* samples (+84%, +67%, and +280% in boiled, steamed, and MW-cooked samples, respectively). Moreover, it was noticed the remarkable increase (+137%) in the content of the unidentified I-der. produced by steaming in *B. vulgaris* (Figure 3).

As for L-and A-der., they showed a very low resistance to boiling but a good resistance to steaming, better than to MW-cooking. In fact, the content of L-der.1 in *A. lutea* steamed samples showed a remarkable increase (+41%). In addition, L-7-gluc. showed a good stability to steaming in *A. lutea*, *H. echioides*, *S. oleraceus*, and *T. officinale*, while its content strongly decreased (−66%) as well as markedly increased (+85%) in MW-cooked samples of *A. lutea* and *S. oleraceus*, respectively. Furthermore, A-7 gluc. showed a better stability to steaming rather than to MW-coking in all species in which they were detected (i.e., *A. lutea*, *B. vulgaris*, and *S. oleraceus*).

Finally, it was noticed that the main unknown compound detected in *A. lutea* (i.e., unknown 1) showed a strong stability to steaming, contrary to boiling and MW-cooking (Figure 3).

In general, changes in phenolic content in vegetables after cooking might mainly result from three sets of processes: (i) the oxidative degradation of phenolic acids (including enzymatic browning), (ii) the release of free acids from conjugated forms, and (iii) the formation of complex structures of phenolic substances from related compounds, such as proteins, tannins and anthocyanins. Depending on the relative intensities of these reactions, the final effect is a decrease (up to a complete loss of CHLA in boiled carrots) or an increase (up to +824% in boiled millet) of phenolic acids [18]. In green asparagus, Sergio et al. [39] found that MW-cooking produced an increase in Q-3-rut. content, while boiling did not cause significant changes in this parameter. It was reported by Vallejo et al. [40] that conventional boiling led to a significant loss of flavonoids (−66%) from fresh broccoli, while high-pressure boiling caused considerable leaching (−47%) of caffeoylquinic acid derivatives into the cooking water; on the other hand, steaming produced minimal loss of both flavonoids and hydroxycynnamoyl derivatives, whereas high losses of flavonoids (−97%), sinapic acid derivatives (−74%) and caffeoylquinic acid derivatives (−87%) were produced by MW-cooking. In partial disagreement with these results, for fresh broccoli Pellegrini et al. [41] reported remarkable increases in the CHLA content after boiling (about fourfold) and oven steaming (about sevenfold), and a significant decrease (about −75%) after MW-cooking.

Therefore, the present results, together with numerous data present in literature, suggested that changes in the content of individual phenolics due to cooking processes essentially depend on two opposite phenomena: (i) the thermal degradation and the water leaching, which reduce their concentration in vegetable tissues, and (ii) a matrix softening effect, which increases their extractability resulting in a higher concentration with respect to raw vegetables [18].

### 3.5. The Effect of Cooking on Antioxidant Activity

The TAA values in the raw wild greens ranged from 3.2 ± 0.3 g Trolox 100 g^−1^ DM in *U. picroides* to 0.6 ± 0.1 g Trolox 100 g^1^ DM in *H. echioides*, with significant differences among species (data not shown).

**Figure 4:**
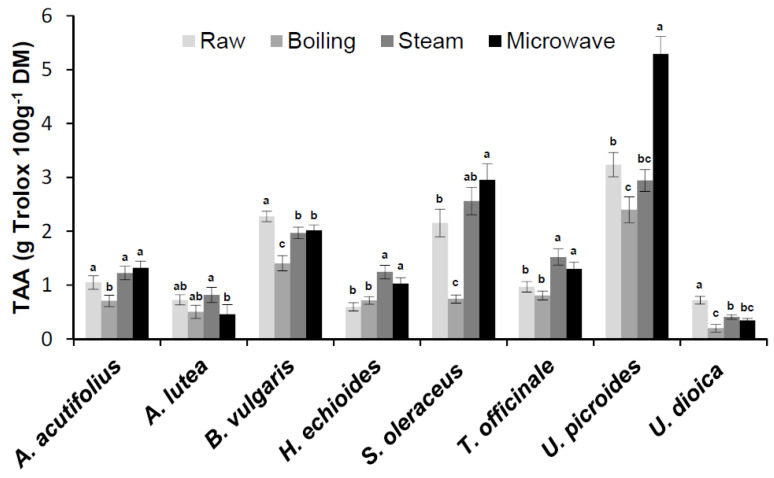
Total antioxidant activity (TAA) in raw and processed wild edible greens. Values indicate the mean ± SD (*n* = 6). Different letters indicate significant differences (*p* < 0.05) among cooking treatments for each species.

The effect of different cooking methods on TAA was shown in Figure 4. As for the cooked greens belonging to the Asteraceae family, after MW-cooking TAA increased in all species while, after steaming, it increased only in *H. echioides* and *T. officinale*, and remained quite unchanged in *S. oleraceus* and *U. picroides*; on the contrary, after boiling it decreased in the two latter species and did not significantly changed in the first two mentioned (Figure 3). In MW-cooked *U. picroides* samples, a very strong TAA increase (+64%) was noticed with respect to the raw vegetable. Moreover, no significant changes in TAA were observed with all cooking procedures in species belonging to Liliaceae family (i.e., *A. acutifolius* and *A. lutea*). In *B. vulgaris* and *U. dioica*, TAA remarkably decreased after boiling (−38% and −72%, respectively). In addition, steaming as well as MW-cooking also produced remarkable TAA decrease (−43% and −52%, respectively) in *U. dioica.*

Findings about the substantial stability of TAA in *H. echioides* after boiling, which resulted in good correlation with the corresponding TPC, were in disagreement with data previously reported by Savo et al. [20]. However, a conceivable explanation has been already given and above discussed (*cfr* Section 3.3).

The results on changes in TAA after cooking were in good agreement with findings previously reported on the same species [19]. Only in the case of *U. picroides* were different results for steaming and MW-cooking found in the two experimental studies. In fact, a significant decrease in TAA values was reported by Boari et al. [19] whatever the cooking procedure used. On the contrary, in the present work, a TAA decrease was found only after boiling, TAA remaining quite unchanged after steaming and increasing very strongly after MW-cooking. Such strong TAA increment caused by MW treatment resulted in good accordance with the significant increase in phenolic content, both in TPC (Table 3) as well as in CHLA and Q-3-rut. content (Figure 3).

A plausible explanation of such different results might be a possible difference in the tissue texture of plants used in the two experimental studies (e.g., caused by different environmental or agronomical factors during the plant growth). Actually, the texture of the vegetable tissue plays an important role in regulating the availability of biochemical soluble nutrients of a vegetable undergoing to cooking, as reported by Renna et al. [38] or the different TPC and TAA stability in relation to boiling shown by two local varieties (“Galatina” and “Molfettese”) of chicory (*Cichorium intybus* L., Catalogna group).

On the other hand, several studies reported changes in TAA in commonly consumed vegetables after cooking, with a great variability of results, often also between the same species. Pellegrini et al. [41] found remarkable increase in antioxidant capacity of broccoli, Brussel sprouts, and cauliflower, particularly relevant in the second one. A decrease in TAA values in artichoke heads blanched in a boiling acidic water solution was reported by Sergio et al. [42]. Moreover, Fanasca et al. [43] reported a significant increase with boiling in TAA values in four cultivars of green asparagus, while Mazzeo et al. [44] found that steaming was able to preserve TAA of several vegetables, in particular increasing it in spinach. Finally, Renna et al. [38] reported an increase in TAA values in MW-cooked stems of two local variety of chicory. In general, steaming and MW resulted the best TAA-preserving cooking methods since, in general, the use of large amounts of water is not recommended to prepare cooking vegetables [18].

### 3.6. The Whole Impact of Cooking on Antioxidant Properties of the Wild Edible Greens

Table 4 shows the correlations between TAA and phenolics for each cooking method in the eight studied species. Highly significant correlations were found between TAA and TPC for each cooking method. As for correlations between TAA and individual phenolic compounds, they resulted significant for: (i) CHLA in boiling and MW-cooking; (ii) CFTA in boiling; (iii) A-7-gluc. in steaming and MW-cooking; the sum of CA-der. (i.e., CHLA+CHCA+ CFTA+CA-der. 1,2,…n) in all cooking methods; and (iv) the sum of flavonoids in boiling and MW-cooking (Table 4). In particular, it was noticed the highly significant correlations between TAA and L-7-gluc. or the sum of CA der., thus confirming the high antioxidant capacity of polyphenols containing catechol groups (i.e., ortho-diphenols) compared with those that have a single hydroxyl group on the aromatic ring [1].

In a similar manner to findings described in the present study, good correlations between TAA and polyphenol content as affected by cooking have been reported for other vegetables. In effect, it has been reported that in four green asparagus cultivars the boiling treatment caused an increase in TAA, total phenols, quercetin, and rutin content [43]. Moreover, in artichoke it caused an increase in the TPC, CA, CHLA, and cynarin contents as well as TAA [45]. It has been also reported that in carrot, cauliflower and spinach steaming produced an increase in polyphenol content, whereas boiling leads to a general loss of phytochemical compounds [44]. Furthermore, the antioxidant activity of steamed or MW-cooked vegetables was generally higher than in boiled samples, because these cooking methods do not produce the release of antioxidants from cooked tissues [18,19,38].

Changes in TAA occurring after cooking depend on many factors: the physical structure of vegetables, the cooking method, the cooking temperature, the chopping extent, the stability of the structure to heating and reactions occurring in the assayed system. In a general way, the phenol amount after cooking results from a balance between two processes: the release of components from the tissue, due to the thermal destruction of cell walls and sub-cellular compartments, and the breakdown of the released compounds. Moreover, boiling may decrease the antioxidant activity by decreasing ascorbic acid, while higher activity may occur as a consequence of the inactivation of oxidative enzymes such as ascorbate oxidase, that also reduces the browning potential [18].

An increase in antioxidant activity was recorded in particular in steamed and MW-cooked samples of the four species belonging to the *Asteraceae* family, this result being supported by a corresponding increase in TPC as well as in the content of some specific phenolic compounds (i.e., CHLA, CHCA, L-7-gluc., and Q-3-rut.). Actually, results indicated that in particular phenolics showed a great stability as well as the tendency to be more available for extraction after steaming and MW-cooking in more than one species. This finding might be very useful for planning new nutritional strategies with innovative food preparation containing fresh as well as cooked wild greens. In fact, the importance in the human diet of hydroxycinnamic acids such as CHLA and CHCA as well as flavonoids such as luteolin and quercetin derivatives is well known [1].

Therefore, within such complex scenario, present results showed that, in the case of the studied species, steaming and MW-cooking proved milder technologies than boiling, thus producing a reduced phenolic loss. In addition, in some species (i.e., *H. echioides*, *S. oleraceus*, and *U. picroides*) such mild treatments (in particular MW-cooking) produced an increase in phenolic content and antioxidant capacity, thus enhancing their nutraceutical value.

## 4. Conclusions

It is difficult to generalize the effect of cooking on phenolic compounds and antioxidant activity because many factors influence the evolution of these parameters. In actuality, cooking facilitates the release of polyphenols that become easily available, but on the other hand the thermal treatment can cause their degradation and, in the case of boiling, a loss due to leaching.

For the first time, the present work evaluated changes in individual phenolic compounds of some wild edible greens in relation to different cooking procedures. Nutritional characteristics and antioxidant potential shown by these species, also after cooking, suggest a reconsideration of their role in the ordinary diet.

Indeed, the trials showed that steaming and MW-cooking are milder technologies than boiling, reducing the phenolic loss. In addition, in some species (i.e., *H. echioides*, *S. oleraceus*, and *U. picroides*) such mild treatments (in particular MW-cooking) increased phenolic content and antioxidant capacity. Therefore, an appropriate cooking method can not only preserve, but also enhance, the nutraceutical value of such vegetables, promoting the increase in some important antioxidants. For these reasons, a growing consumption of such underutilized species is desirable, as cooked vegetables as well as processed food in new dishes and recipes.

## Figures and Tables

**Table 1:** Nomenclature and utilization of the studied wild greens.

Scientific Name	Family Name	Common Name	Used Part	Traditional Uses
*Asparagus acutifolius* L.	Liliaceae	Wild Asparagus	Stems	Boiled, fried, omelets, soup, pickles, in oil
*Asphodeline lutea* (L.) Rchb.	Liliaceae	Yellow Asfodel	Stems, roots	Salad, boiled, fried, grilled, omelets
*Beta vulgaris* subsp. *maritima* (L.) Arcang.	Chenopodiaceae	Common Beet	Leaves, stems	Salad, boiled, vegetable pie
*Helminthotheca echioides* L.	Asteraceae	Bristy Ox-Tongue	Leaves, stems, roots	Salad, boiled
*Sonchus oleraceus* L.	Asteraceae	Sow Thistle	Leaves, stems, flowers	Salad, boiled, soup, vegetable pie
*Taraxacum officinale* Weber	Asteraceae	Common Dandelion	Leaves, roots, flowers, germ	Salad, boiled, fried, soup, vegetable pie, pickles, in oil
*Urospermum picroides* (L.) Schmidt	Asteraceae	Prickly Golden Fleece	Leaves, stems	Boiled
*Urtica dioica* L.	Urticaceae	Stinging Nettle	Leaves, stems, flowers	Boiled, fried, omelets, soup, vegetable pie

**Table 2:** Phenolic compound identity and content of raw wild edible greens evaluated by HPLC-DAD. Retention times of all peaks have been normalized to those of caffeic acid (CA) as an internal standard (I.S.). Values indicate the mean ± SD (*n* = 6).

Peak Number	Retention Time ^(a)^ (min)	λ_max_^(b)^ (nm)	Phenolic Compound Identity ^(c)^	Phenolic Compound Content (mg 100g ^−1^ DM)							
				Species							
				*Asparagus acutifolius*	*Asphodeline lutea*	*Beta vulgaris*	*Helminthotheca echioides*	*Sonchus oleraceus*	*Taraxacum officinale*	*Urospermum picroides*	*Urtica dioica*
1	5.15	326	neo-CHLA								56 ± 7
2	5.81	275	unknown 1		172 ±13						
3	5.87	327	CFTA				63 ± 5	109 ± 11	114 ± 10		
4	8.18	326	CHLA	55 ± 5			23 ± 3	66 ± 8	29 ± 3	1355 ± 148	563 ± 67
5	8.29	303	unknown 2		18 ± 3						
6	9.80	326	CA-der. 1	12 ± 3	14 ± 4						
7	10.80	325	CA (I.S.)				tr.^(d)^	tr.^(d)^	tr.^(d)^	tr.^(d)^	
8	11.77	315	unknown 3	15 ± 4							
9	12.71	327	CA-der. 2				tr.^(d)^	tr.^(d)^	28 ± 4	tr.^(d)^	
10	13.93	325	CA-der. 3	18 ± 5							
11	16.45	331	CHCA	65 ± 7			301 ± 42	725 ± 85	625 ± 72		
12	16.93	255; 349	L-der. 1		50 ± 4						
13	18.00	267; 344	unknown 4		17 ± 3						
14	20.01	269; 338	A-der. 1			1600 ± 192					
15	21.31	269; 338	A-der. 2			115 ± 9					
16	21.20	326	3,5-DCQA	11 ± 4			25 ± 2	64 ± 5	40 ± 5	110 ± 13	
17	22.25	326	1,5-DCQA	14 ± 5							
18	22.53	256; 351	L-7-glucoside		149 ± 18		115 ± 12	444 ± 51	11 ± 3		
19	22.92	257; 356	Q-3-glucoside							412 ± 48	
20	23.65	257; 356	Q-3-rutinoside	299 ± 27						147 ± 16	
21	24.96	269; 337	A-der. 3			672 ± 55					
22	25.56	257; 356	Q-der.							133 ± 18	tr.^(d)^
23	25.90	256; 352	I-der.			122 ± 15					
24	27.50	317	unknown 5	28 ± 5							
25	27.67	268; 339	A-7-glucoside		19 ± 3	59 ± 8		544 ± 67			
26	28.33	255; 349	L-der. 2								18 ± 4
27	28.83	266; 349	K-3-rutinoside	18 ± 4							
28	29.85	256; 351	L-der. 3		21 ± 5						
29	29.97	255; 356	I-3-rutinoside	215 ± 18							
30	39.66	255; 351	L-der. 4		69 ± 8						
31	43.88	317	unknown 6	64 ± 10							
32	44.02	319	unknown 7		35 ± 4						
33	45.32	303	unknown 8						29 ± 2		
34	50.38	275	unknown 9		36 ± 4						

^(a)^ retention time normalized to caffeic acid as internal standard (I.S.); ^(b)^ wavelength/s of the maximum/a of absorbance shown by the UV-vis spectrum of the given peak in the range 250–600 nm; ^(c)^ der. = derivative; CHLA = chlorogenic acid; CFTA = caftaric acid; CA = caffeic acid; CHCA = chicoric acid; A = apigenin; I = isorhamnetin; K = kaempferol; L = luteolin; Q = quercetin; DCQA = di-caffeoylquinic acid; ^(d)^ tr. = traces (i.e., phenolic compound content below the limit of quantification).

**Table 3:** Total phenolic content (TPC) in wild edible greens. Values indicate the mean ± SD (*n* = 6).

Species	Total Phenolic Content (TPC) (mg CAE 100g^−1^ DM)			
	Raw	Boiling	Steam	Microwave
*A. acutifolius*	1200 ± 57 ^b,A^	873 ± 33 ^d,B^	1196 ± 60 ^d,A^	1136 ± 52 ^c,A^
*A. lutea*	1055 ± 52 ^c,A^	756 ± 42 ^d,B^	955 ± 60 ^e,A^	777 ± 53 ^d,B^
*B. vulgaris*	2477 ± 255 ^a,A^	1443 ± 81 ^b,B^	2375 ± 108 ^b,A^	1962 ± 264 ^b,AB^
*H. echioides*	810 ± 115 ^d,C^	1410 ± 117 ^bc,AB^	1184 ± 124 ^d,B^	1495 ± 268 ^c,A^
*S. oleraceus*	1394 ± 231 ^b,B^	644 ± 44 ^de,C^	2005 ± 212 ^c,A^	2371 ± 472 ^b,A^
*T. officinale*	1044 ± 265 ^bcd,A^	1108 ± 212 ^c,A^	1315 ± 210 ^d,A^	1151 ± 192 ^c,A^
*U. picroides*	2574 ± 247 ^a,BC^	2109 ± 245 ^a,C^	2811 ± 152 ^a,B^	4632 ± 104 ^a,A^
*U. dioica*	735 ± 87 ^d,A^	184 ± 65 ^f,C^	443 ± 73 ^f,B^	326 ± 75 ^e,BC^

CAE = caffeic acid equivalent; DM = dry matter. Different small letters indicate significant differences (*p* < 0.05) within columns; Different capital letters indicate significant differences (*p* < 0.05) within rows.

**Table 4:** Significance of correlations between total antioxidant activity (TAA) and total phenolic content (TPC) or individual phenolic compounds for each cooking treatment in the eight studied species.

Cooking Method	TPC	Chlorogenic Acid	Chicoric Acid	Caftaric Acid	Apigenin-7-Glucoside	Luteolin-7-Glucoside	Sum of Caffeic Acid Derivatives	Sum of Flavonoids
**Raw**	**	ns	ns	ns	ns	***	*	ns
**Boiling**	***	**	ns	*	ns	ns	*	*
**Steam**	***	ns	ns	ns	*	***	**	ns
**Microwave**	****	**	ns	ns	*	***	**	*

Significance: * = significant for *P* < 0.05, ** = significant for *P* < 0.01, *** = significant for *P* < 0.001, **** = significant for *P* < 0.000; ns = not significant.
